# *trans*-Dichlorido(1,4,8,11-tetra­azacyclo­tetra­deca­ne)manganese(III) tetra­fluorido­borate

**DOI:** 10.1107/S1600536810004058

**Published:** 2010-02-06

**Authors:** Donia Zaouali Zgolli, Habib Boughzala, Ahmed Driss

**Affiliations:** a Laboratoire de Matériaux et Cristallochimie, Faculté des Sciences, El Manar, 2092 Tunis, Tunisia

## Abstract

In the title manganese(III) complex, [MnCl_2_(C_10_H_24_N_4_)]BF_4_ or *trans*-[MnCl_2_(cyclam)]BF_4_ (cyclam is the tetra­dentate amine ligand 1,4,8,11-tetra­azacyclo­tetra­deca­ne), the Mn^III^ ions occupy the center of a distorted octa­hedron coordinated by all four ligand nitro­gen donors in the macrobicyclic cavity and two chloride ions occupy the axial positions. Intra­molecular hydrogen bonding involving the coordinated chloride ions and the hydrogen atoms of the cyclam ligand is observed. Inter­molecular hydrogen bonding involving the tetrafluoridoborate anion and hydrogen atoms of the cyclam ligand leads to an infinite one-dimensional chain along the *a* axis. The tetra­fluoridoborate and inorganic units are linked by N—H⋯F hydrogen bonds. The structure may be compared with those of analogous compounds [MnCl_2_(cyclam)]ClO_4_ and [Mn(CN)_2_(cyclam)]ClO_4_.

## Related literature

For applications of cyclams, see: Lindoy (1992 ▶); Izatt *et al.* (1991 ▶, 1995 ▶); Enoki *et al.* (2003 ▶); Steward & McLaughlin (2004 ▶); Sibert (2002 ▶); Volkert & Hoffman (1999 ▶); Anderson & Welch (1999 ▶); Caravan *et al.* (1999 ▶). For isostructural compounds, see: Shaikh *et al.* (2004 ▶); Mossin *et al.* (2002 ▶). For other cyclam-containing structures, see: Brewer *et al.* (1989 ▶); Letumier *et al.* (1998 ▶); Bakac & Espenson (1987 ▶); Mossin *et al.* (2005 ▶); Blessing (1987 ▶); Sosa-Torres & Toscano (1997 ▶).
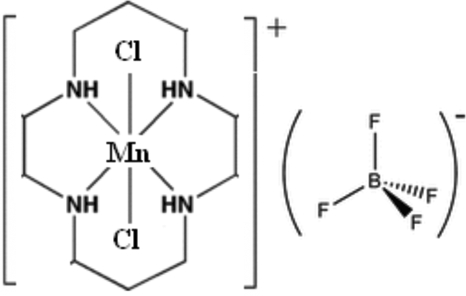


## Experimental

### 

#### Crystal data

[MnCl_2_(C_10_H_24_N_4_)]BF_4_
*M* *_r_* = 412.98
Orthorhombic, 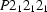

*a* = 6.5660 (3) Å
*b* = 13.3760 (2) Å
*c* = 19.5846 (3) Å
*V* = 1720.05 (9) Å^3^
*Z* = 4
Mo *K*α radiation
μ = 1.12 mm^−1^
*T* = 298 K
0.40 × 0.40 × 0.20 mm

#### Data collection

Enraf–Nonius CAD-4 diffractometer
Absorption correction: ψ scan (North *et al.*, 1968 ▶) *T*_min_ = 0.674, *T*_max_ = 0.814
3013 measured reflections
2735 independent reflections
2442 reflections with *I* > 2σ(*I*)
*R*_int_ = 0.020
2 standard reflections every 120 min  intensity decay: 3%

#### Refinement

*R*[*F* ^2^ > 2σ(*F* ^2^)] = 0.034
*wR*(*F* ^2^) = 0.097
*S* = 1.05
2735 reflections
199 parameters
H-atom parameters constrained
Δρ_max_ = 0.70 e Å^−3^
Δρ_min_ = −0.50 e Å^−3^
Absolute structure: Flack (1983 ▶), 569 Friedel pairs
Flack parameter: 0.00 (3)

### 

Data collection: *CAD-4 EXPRESS* (Enraf–Nonius, 1994 ▶); cell refinement: *CAD-4 EXPRESS*; data reduction: *XCAD4* (Harms & Wocadlo, 1995 ▶); program(s) used to solve structure: *SHELXS97* (Sheldrick, 2008 ▶); program(s) used to refine structure: *SHELXL97* (Sheldrick, 2008 ▶); molecular graphics: *ORTEP-3 for Windows* (Farrugia, 1997 ▶); software used to prepare material for publication: *WinGX* (Farrugia, 1999 ▶).

## Supplementary Material

Crystal structure: contains datablocks I, global. DOI: 10.1107/S1600536810004058/br2133sup1.cif

Structure factors: contains datablocks I. DOI: 10.1107/S1600536810004058/br2133Isup2.hkl

Additional supplementary materials:  crystallographic information; 3D view; checkCIF report

## Figures and Tables

**Table 1:** Hydrogen-bond geometry (Å, °)

*D*—H⋯*A*	*D*—H	H⋯*A*	*D*⋯*A*	*D*—H⋯*A*
N1—H1⋯F4^i^	0.91	2.24	3.025 (6)	145
N2—H2⋯Cl1^ii^	0.91	2.44	3.256 (3)	149
N3—H3⋯F3^ii^	0.91	2.34	3.116 (5)	143
N4—H4⋯Cl2^iii^	0.91	2.49	3.289 (3)	147

Symmetry codes: (i) 
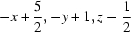
; (ii) 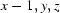; (iii) 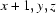.

**Fig. 1.**
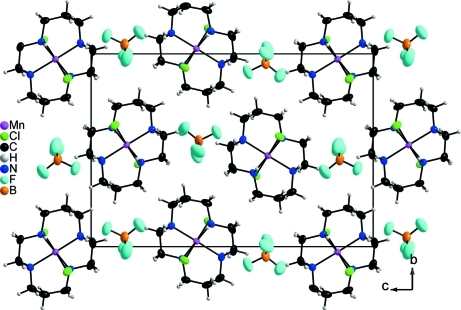
Projection in bc plane of [MnCl2(cyclam)]BF4 crystal structure. Displacement ellipsoids are drawn at the 50% probability level.

**Fig. 2.**
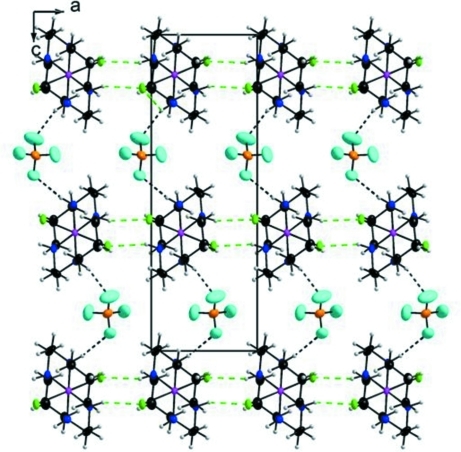
Molecular packing diagram of [MnCl2(cyclam)]BF4 showing intra- and intermolecular hydrogen bonding interaction giving rise to infinite one-dimensional chain. Ellipsoids are drawn at the 50% probability level.

## supplementary crystallographic information

### Comment

Cyclam-based metal complexes are attractive for many applications (Lindoy (1992), Izatt *et al.* (1991 and 1995)). Phenylazomethine dendrimers with a cyclam core have been found being able to form multinuclear hetero-metal complexes (Enoki *et al.* (2003)). Four arm oligonucleotide Ni–cyclam complexes can form highly ordered lattices with exceptional structural, electric and photoelectric properties (Steward *et al.* (2004)). Recently, it was found that lipophilic cyclams possess anti-tumour activity (Sibert *et al.*, 2002). Furthermore, cyclam derivatives have been studied extensively as possible agents for magnetic resonance imaging (Caravan *et al.* (1999)), radiodiagnostic imaging (Anderson *et al.* (1999)) and therapeutic radiopharmaceuticals (Volkert *et al.* (1999)). In this paper, we report the synthesis and single-crystal X-ray diffraction studies of the organic-inorganic hybrid compound: [MnCl_2_(cyclam)].BF_4 _(a).

The title compound (a) is isostructural to the structure of the [MnCl_2_(cyclam)].ClO_4 _(b) and [Mn(CN)_2_(cyclam)]ClO_4 _(c) reported by Shaikh *et al.* (2004) and Mossin *et al.* (2002) respectively. In three molecules, the asymmetric unit contains an inorganic cation Zcyclam manganese (III) (Z: Cl (a and b), CN (c)) and A*X*_4_ anion (A*X*_4_: tetrafluoridoborate (BF_4_) (a), perchlorate (ClO_4_) (b and c). They have the same space group (*P*2_1_2_1_2_1_) and they are characterized by one-dimensional hydrogen-bonded networks.

The substitution of two chlorine atoms in (b) by two cyano atoms in (c) appears to have the same unit cell. This is probably indicative of the same size and same charge of the two ligands.

The title compound, [Mn(cyclam)Cl_2_].BF_4_ is constructed from isolated Mn(cyclam)Cl_2_ octahedra and the tetrafluoridoborate molecules (BF_4_)(Fig 1).  The manganese atom is octahedrally coordinated to four N atoms of the macrocycle in the basal position and two chlorine atoms in axial positions. The bond lengths in the title compound are shorter than those found for the corresponding bonds in the salts of [Mn(cyclam)O]^2^_2_ which is probably indicative of the *trans*-influence of oxygen in the later ion (Brewer *et al.* (1989)). The average Mn–N distance here (2.051 (3) Å) is slightly higher than that (2.033 Å) (Table 2) observed in *trans*-[Mn(cyclam) Cl_2_]Cl.5H_2_O (Letumier *et al.* (1998)). This bond distance (Mn–Cl) is much longer than that found in the similar cation [CoCl_2_(cyclam)]^+^ where the Co–Cl distance is 2.252 Å (Bakac *et al.*(1987)). The N–Mn–N bond angles, like the bond distances and angles in the cyclam ligand, are thoroughly consistent with those in the literature (Sosa-Torres *et al.* (1997), Blessing (1987) and Mossin *et al.* (2005)).

The protonated cation and the deprotonated anion are linked through a number of intramolecular N—H···Cl and intermolecular N—H···F hydrogen bonds (Fig 2)

These ligands (cyclam) provide interesting new possibilities in the field of the treatment of waste water contaminated by toxic or radioactive metals and gas purification since they can be attached to an organic or inorganic solid support *via* relatively simple reactions. In this respect, thermodynamic and magnetic data are complementary and most useful information which will allow rational design by the molecular engineering of more efficient chelating agents.

### Experimental

The title compound [Mn(cyclam)Cl_2_].BF_4_ was prepared from methanol solution containing 1,4,8,11-Tetraazacyclotetradecane (cyclam) and stoichiometric amount of manganese chloride (MnCl_2_.4H_2_O) under hydrofluoric acid (HF) conditions. The resulting mixture was heated to boiling point and stirred for two hours. After several weeks single green crystals were obtained by slow evaporation from aqueous solution at room temperature. Boron was diffused from the Pyrex crystallizing (13% borosilicate).

### Refinement

All H atoms attached to C atoms and N atoms were fixed geometrically and treated as riding with C—H = 0.97Å and N—H = 0.91 Å.

### Crystal data

**Table:**

[MnCl_2_(C_10_H_24_N_4_)]BF_4_	*F*(000) = 848
*M**_r_* = 412.98	*D*_x_ = 1.59 Mg m^−^^3^
Orthorhombic, *P*2_1_2_1_2_1_	Mo *K*α radiation, λ = 0.71073 Å
Hall symbol: P 2ac 2ab	Cell parameters from 25 reflections
*a* = 6.5660 (3) Å	θ = 10–15°
*b* = 13.3760 (2) Å	µ = 1.12 mm^−^^1^
*c* = 19.5846 (3) Å	*T* = 298 K
*V* = 1720.05 (9) Å^3^	Prism, green
*Z* = 4	0.40 × 0.40 × 0.20 mm

### Data collection

**Table:**

Enraf–Nonius CAD-4 diffractometer	2442 reflections with *I* > 2σ(*I*)
Radiation source: fine-focus sealed tube	*R*_int_ = 0.020
graphite	θ_max_ = 27.0°, θ_min_ = 2.1°
non–profiled ω/2θ scans	*h* = −8→2
Absorption correction: ψ scan (North *et al.*, 1968)	*k* = −1→17
*T*_min_ = 0.674, *T*_max_ = 0.814	*l* = −1→24
3013 measured reflections	2 standard reflections every 120 min
2735 independent reflections	intensity decay: 3%

### Refinement

**Table:**

Refinement on *F*^2^	Secondary atom site location: difference Fourier map
Least-squares matrix: full	Hydrogen site location: inferred from neighbouring sites
*R*[*F*^2^ > 2σ(*F*^2^)] = 0.034	H-atom parameters constrained
*wR*(*F*^2^) = 0.097	*w* = 1/[σ^2^(*F*_o_^2^) + (0.0545*P*)^2^ + 1.0781*P*] where *P* = (*F*_o_^2^ + 2*F*_c_^2^)/3
*S* = 1.05	(Δ/σ)_max_ < 0.001
2735 reflections	Δρ_max_ = 0.70 e Å^−^^3^
199 parameters	Δρ_min_ = −0.50 e Å^−^^3^
0 restraints	Absolute structure: Flack (1983), 569 Friedel pairs
Primary atom site location: structure-invariant direct methods	Flack parameter: 0.00 (3)

### Special details

**Table:**

Experimental. Absorption correction: Number of psi-scan sets used was 5 Theta correction was applied. Averaged transmission function was used. No Fourier smoothing was applied.
Geometry. All e.s.d.'s (except the e.s.d. in the dihedral angle between two l.s. planes) are estimated using the full covariance matrix. The cell e.s.d.'s are taken into account individually in the estimation of e.s.d.'s in distances, angles and torsion angles; correlations between e.s.d.'s in cell parameters are only used when they are defined by crystal symmetry. An approximate (isotropic) treatment of cell e.s.d.'s is used for estimating e.s.d.'s involving l.s. planes.
Refinement. Refinement of *F*^2^ against ALL reflections. The weighted *R*-factor *wR* and goodness of fit *S* are based on *F*^2^, conventional *R*-factors *R* are based on *F*, with *F* set to zero for negative *F*^2^. The threshold expression of *F*^2^ > σ(*F*^2^) is used only for calculating *R*-factors(gt) *etc*. and is not relevant to the choice of reflections for refinement. *R*-factors based on *F*^2^ are statistically about twice as large as those based on *F*, and *R*- factors based on ALL data will be even larger.

### Fractional atomic coordinates and isotropic or equivalent isotropic displacement parameters (Å^2^)

**Table:**

	*x*	*y*	*z*	*U*_iso_*/*U*_eq_	
Mn	0.77998 (8)	0.47206 (4)	0.37355 (2)	0.02124 (13)	
Cl1	1.08555 (14)	0.57065 (7)	0.33104 (5)	0.0340 (2)	
Cl2	0.47956 (13)	0.37068 (8)	0.41610 (5)	0.0350 (2)	
C1	0.6089 (7)	0.4243 (3)	0.24327 (19)	0.0355 (9)	
H1A	0.4871	0.3929	0.2613	0.043*	
H1B	0.6247	0.4045	0.1959	0.043*	
C2	0.5915 (7)	0.5364 (3)	0.24847 (18)	0.0347 (9)	
H2A	0.4700	0.5592	0.2251	0.042*	
H2B	0.7089	0.5679	0.2275	0.042*	
C3	0.6006 (7)	0.6724 (3)	0.3358 (2)	0.0337 (9)	
H3A	0.4972	0.7077	0.3099	0.040*	
H3B	0.7328	0.6948	0.3198	0.040*	
C4	0.5780 (7)	0.6979 (3)	0.4115 (2)	0.0401 (10)	
H4A	0.4539	0.6674	0.4284	0.048*	
H4B	0.5633	0.7698	0.4160	0.048*	
C5	0.7560 (7)	0.6637 (3)	0.4564 (2)	0.0393 (9)	
H5A	0.8826	0.6880	0.4371	0.047*	
H5B	0.7413	0.6927	0.5016	0.047*	
C6	0.9408 (7)	0.5187 (4)	0.50530 (19)	0.0403 (10)	
H6A	0.9178	0.5363	0.5527	0.048*	
H6B	1.0654	0.5508	0.4903	0.048*	
C7	0.9609 (7)	0.4063 (4)	0.4985 (2)	0.0409 (11)	
H7A	1.0804	0.3831	0.5230	0.049*	
H7B	0.8420	0.3736	0.5176	0.049*	
C8	0.9738 (7)	0.2730 (3)	0.4095 (2)	0.0405 (10)	
H8A	0.8453	0.2457	0.4252	0.049*	
H8B	1.0824	0.2398	0.4343	0.049*	
C9	0.9975 (8)	0.2522 (3)	0.3335 (3)	0.0445 (11)	
H9A	1.0221	0.1813	0.3273	0.053*	
H9B	1.1168	0.2876	0.3171	0.053*	
C10	0.8144 (7)	0.2825 (3)	0.2895 (2)	0.0388 (10)	
H10A	0.8304	0.2544	0.2442	0.047*	
H10B	0.6914	0.2546	0.3093	0.047*	
N1	0.7920 (5)	0.3928 (2)	0.28397 (15)	0.0283 (6)	
H1	0.9032	0.4152	0.2609	0.034*	
N2	0.5806 (5)	0.5632 (2)	0.32309 (14)	0.0245 (6)	
H2	0.4538	0.5456	0.3375	0.029*	
N3	0.7654 (5)	0.5538 (2)	0.46247 (14)	0.0294 (7)	
H3	0.6498	0.5344	0.4844	0.035*	
N4	0.9798 (5)	0.3817 (2)	0.42444 (16)	0.0271 (7)	
H4	1.1063	0.4026	0.4118	0.032*	
F1	1.2555 (6)	0.4764 (4)	0.62517 (17)	0.0960 (13)	
F2	1.5921 (6)	0.4961 (3)	0.62145 (19)	0.0869 (12)	
F3	1.3893 (6)	0.5936 (3)	0.55560 (14)	0.0730 (10)	
F4	1.3937 (9)	0.6116 (3)	0.67105 (19)	0.1155 (18)	
B1	1.4102 (9)	0.5490 (5)	0.6183 (3)	0.0480 (13)	

### Atomic displacement parameters (Å^2^)

**Table:**

	*U*^11^	*U*^22^	*U*^33^	*U*^12^	*U*^13^	*U*^23^
Mn	0.0161 (2)	0.0242 (2)	0.0234 (2)	0.0004 (2)	−0.0017 (2)	0.0004 (2)
Cl1	0.0189 (4)	0.0365 (5)	0.0467 (5)	−0.0043 (4)	0.0023 (4)	0.0101 (4)
Cl2	0.0190 (4)	0.0414 (5)	0.0445 (5)	−0.0054 (4)	0.0022 (4)	0.0095 (4)
C1	0.033 (2)	0.046 (2)	0.0278 (17)	−0.005 (2)	−0.0042 (17)	−0.0046 (18)
C2	0.032 (2)	0.044 (2)	0.0276 (17)	0.000 (2)	−0.0093 (17)	0.0073 (17)
C3	0.030 (2)	0.0259 (18)	0.045 (2)	0.0040 (18)	−0.002 (2)	0.0064 (16)
C4	0.034 (2)	0.0282 (19)	0.058 (2)	0.0078 (19)	0.001 (2)	−0.0099 (18)
C5	0.037 (2)	0.0372 (19)	0.044 (2)	−0.002 (2)	−0.002 (2)	−0.0129 (17)
C6	0.034 (2)	0.060 (3)	0.0263 (18)	−0.005 (2)	−0.0107 (17)	−0.004 (2)
C7	0.031 (2)	0.056 (3)	0.035 (2)	−0.004 (2)	−0.0072 (18)	0.015 (2)
C8	0.032 (2)	0.030 (2)	0.059 (3)	0.0023 (19)	−0.001 (2)	0.015 (2)
C9	0.040 (2)	0.028 (2)	0.066 (3)	0.004 (2)	0.004 (2)	−0.006 (2)
C10	0.035 (2)	0.0306 (19)	0.051 (2)	−0.0004 (19)	0.003 (2)	−0.0123 (17)
N1	0.0253 (15)	0.0300 (14)	0.0295 (13)	−0.0021 (15)	0.0012 (15)	−0.0027 (11)
N2	0.0179 (13)	0.0265 (14)	0.0291 (14)	−0.0010 (13)	0.0000 (13)	0.0033 (12)
N3	0.0212 (15)	0.0399 (16)	0.0271 (12)	−0.0027 (15)	0.0024 (13)	−0.0055 (12)
N4	0.0179 (13)	0.0304 (16)	0.0330 (15)	−0.0017 (14)	−0.0018 (13)	0.0075 (13)
F1	0.069 (2)	0.143 (4)	0.076 (2)	−0.037 (3)	−0.004 (2)	0.026 (2)
F2	0.0534 (19)	0.094 (3)	0.113 (3)	0.0194 (19)	−0.014 (2)	0.024 (2)
F3	0.065 (2)	0.102 (3)	0.0516 (15)	0.001 (2)	−0.0040 (16)	0.0232 (17)
F4	0.166 (5)	0.104 (3)	0.076 (2)	0.041 (4)	−0.031 (3)	−0.028 (2)
B1	0.043 (3)	0.065 (3)	0.036 (2)	0.011 (3)	−0.009 (2)	0.004 (2)

### Geometric parameters (Å, °)

**Table:**

Mn—N2	2.043 (3)	C6—C7	1.516 (6)
Mn—N4	2.043 (3)	C6—H6A	0.9700
Mn—N1	2.052 (3)	C6—H6B	0.9700
Mn—N3	2.059 (3)	C7—N4	1.492 (5)
Mn—Cl2	2.5346 (10)	C7—H7A	0.9700
Mn—Cl1	2.5412 (10)	C7—H7B	0.9700
C1—N1	1.503 (5)	C8—N4	1.484 (5)
C1—C2	1.506 (6)	C8—C9	1.521 (7)
C1—H1A	0.9700	C8—H8A	0.9700
C1—H1B	0.9700	C8—H8B	0.9700
C2—N2	1.506 (4)	C9—C10	1.533 (6)
C2—H2A	0.9700	C9—H9A	0.9700
C2—H2B	0.9700	C9—H9B	0.9700
C3—N2	1.488 (5)	C10—N1	1.486 (5)
C3—C4	1.529 (6)	C10—H10A	0.9700
C3—H3A	0.9700	C10—H10B	0.9700
C3—H3B	0.9700	N1—H1	0.9100
C4—C5	1.532 (6)	N2—H2	0.9100
C4—H4A	0.9700	N3—H3	0.9100
C4—H4B	0.9700	N4—H4	0.9100
C5—N3	1.476 (5)	F1—B1	1.412 (7)
C5—H5A	0.9700	F2—B1	1.389 (7)
C5—H5B	0.9700	F3—B1	1.373 (6)
C6—N3	1.500 (5)	F4—B1	1.333 (7)
			
N2—Mn—N4	179.62 (14)	C6—C7—H7A	110.1
N2—Mn—N1	85.38 (12)	N4—C7—H7B	110.1
N4—Mn—N1	94.96 (13)	C6—C7—H7B	110.1
N2—Mn—N3	93.58 (12)	H7A—C7—H7B	108.4
N4—Mn—N3	86.07 (13)	N4—C8—C9	111.7 (4)
N1—Mn—N3	178.93 (13)	N4—C8—H8A	109.3
N2—Mn—Cl2	88.83 (9)	C9—C8—H8A	109.3
N4—Mn—Cl2	91.32 (9)	N4—C8—H8B	109.3
N1—Mn—Cl2	91.98 (10)	C9—C8—H8B	109.3
N3—Mn—Cl2	88.27 (9)	H8A—C8—H8B	107.9
N2—Mn—Cl1	92.17 (9)	C8—C9—C10	114.9 (4)
N4—Mn—Cl1	87.68 (9)	C8—C9—H9A	108.5
N1—Mn—Cl1	87.58 (10)	C10—C9—H9A	108.5
N3—Mn—Cl1	92.20 (9)	C8—C9—H9B	108.5
Cl2—Mn—Cl1	178.87 (4)	C10—C9—H9B	108.5
N1—C1—C2	107.7 (3)	H9A—C9—H9B	107.5
N1—C1—H1A	110.2	N1—C10—C9	112.4 (3)
C2—C1—H1A	110.2	N1—C10—H10A	109.1
N1—C1—H1B	110.2	C9—C10—H10A	109.1
C2—C1—H1B	110.2	N1—C10—H10B	109.1
H1A—C1—H1B	108.5	C9—C10—H10B	109.1
C1—C2—N2	107.8 (3)	H10A—C10—H10B	107.9
C1—C2—H2A	110.1	C10—N1—C1	113.4 (3)
N2—C2—H2A	110.1	C10—N1—Mn	117.0 (2)
C1—C2—H2B	110.1	C1—N1—Mn	106.1 (2)
N2—C2—H2B	110.1	C10—N1—H1	106.6
H2A—C2—H2B	108.5	C1—N1—H1	106.6
N2—C3—C4	111.9 (3)	Mn—N1—H1	106.6
N2—C3—H3A	109.2	C3—N2—C2	113.0 (3)
C4—C3—H3A	109.2	C3—N2—Mn	116.6 (2)
N2—C3—H3B	109.2	C2—N2—Mn	107.3 (2)
C4—C3—H3B	109.2	C3—N2—H2	106.4
H3A—C3—H3B	107.9	C2—N2—H2	106.4
C3—C4—C5	114.6 (4)	Mn—N2—H2	106.4
C3—C4—H4A	108.6	C5—N3—C6	112.9 (3)
C5—C4—H4A	108.6	C5—N3—Mn	117.6 (2)
C3—C4—H4B	108.6	C6—N3—Mn	105.7 (2)
C5—C4—H4B	108.6	C5—N3—H3	106.7
H4A—C4—H4B	107.6	C6—N3—H3	106.7
N3—C5—C4	112.0 (3)	Mn—N3—H3	106.7
N3—C5—H5A	109.2	C8—N4—C7	113.9 (3)
C4—C5—H5A	109.2	C8—N4—Mn	117.8 (3)
N3—C5—H5B	109.2	C7—N4—Mn	106.9 (3)
C4—C5—H5B	109.2	C8—N4—H4	105.8
H5A—C5—H5B	107.9	C7—N4—H4	105.8
N3—C6—C7	109.2 (4)	Mn—N4—H4	105.8
N3—C6—H6A	109.8	F4—B1—F3	114.3 (5)
C7—C6—H6A	109.8	F4—B1—F2	110.8 (5)
N3—C6—H6B	109.8	F3—B1—F2	110.3 (5)
C7—C6—H6B	109.8	F4—B1—F1	107.5 (5)
H6A—C6—H6B	108.3	F3—B1—F1	108.2 (4)
N4—C7—C6	108.1 (3)	F2—B1—F1	105.3 (4)
N4—C7—H7A	110.1		

### Hydrogen-bond geometry (Å, °)

**Table:**

*D*—H···*A*	*D*—H	H···*A*	*D*···*A*	*D*—H···*A*
N1—H1···F4^i^	0.91	2.24	3.025 (6)	145
N2—H2···Cl1^ii^	0.91	2.44	3.256 (3)	149
N3—H3···F3^ii^	0.91	2.34	3.116 (5)	143
N4—H4···Cl2^iii^	0.91	2.49	3.289 (3)	147

Symmetry codes: (i) −*x*+5/2, −*y*+1, *z*−1/2; (ii) *x*−1, *y*, *z*; (iii) *x*+1, *y*, *z*.
